# Supplementary material for: Demulsification of Emulsion Using Heptanoic Acid during Aqueous Enzymatic Extraction and the Characterization of Peanut Oil and Proteins Extracted
Source: Foods. 2023 Sep 22;12(19):3523. doi: 10.3390/foods12193523 (PMC10572140; doi:10.3390/foods12193523)
Supplement: Supplementary file 1 [file foods-12-03523-s001.zip › foods-2616777-supplementary.pdf]

Supplementary material

Acid value

Weigh the oil sample (20 g) and place it in a conical bottle. Added ether - isopropyl alcohol mixture (50~100mL) and phenolphthalein indicator (3~4 drops) to sample, shaken and dissolved the sample fully. The standard alkali solution was used to titrate the sample solution. The end point of titration is when the sample solution is slightly red at the beginning and there is no obvious fading within 15s. Immediately stop the titration, record the standard titration solution volume V_1 consumed by this titration, and measure the standard volume V_0 consumed by the blank reagent.

$$\text{Acid value} = \frac{(V_1 - V_0) \times C \times 56.1}{m}$$

V_1- volume, mL of standard alkali used in the titration
V_0- volume, mL of standard alkali used in the titration the bank
C- normality of standard alkali, mol/L
56.1-molar mass of KOH, g/mol
m- mass, grams of sample

Peroxide value

The 2g~3g samples were placed in a 250 mL iodine bottle, 30 mL of trichloromethane and glacial acetic acid mixture were added, and the sample was completely dissolved by gently shaking. Accurately add 1.00 mL of saturated potassium iodide solution, plug the bottle tightly, shake gently for 0.5 minutes, and place in the dark for 3 minutes. Removed and added 100 mL of water, shaken well, immediately titrated the iodine precipitated by standard dissolution titration with sodium thiosulfate, titrated until light yellow, added 1 mL of starch indicator, continued titrating and strongly shaking until the blue color of the solution disappeared as the end point, and recorded the titration volume V_1. At the same time, the blank test was carried out.

$$\text{peroxide value} = \frac{(V_1 - V_0) \times C \times 0.1269}{m} \times 100$$

V_1- volume, mL of standard $Na_2S_2O_3$ used in the titration
V_0- volume, mL of standard $Na_2S_2O_3$ used in the titration the bank
C- normality of standard $Na_2S_2O_3$, mol/L
0.1269- the mass of iodine equivalent to 1.00mL sodium thiosulfate standard titration solution [c(Na2S2O3) =1.000mol/L]
m- mass, grams of sample

Iodine value

According to the expected iodine value of the sample, an appropriate amount of the sample was weighed into the conical bottle, and an equal amount of cyclohexane and glacial acetic acid (φ=50mL/100mL) was added to dissolve the sample. Then, add 25 mL Wijs solution, cover the plug, and shake well. Keep in a dark place for 1 hours. After the reaction is over, add 20mL potassium iodide solution and 150 mL water. Titrate with a calibrated sodium thiosulfate solution until the yellow color of iodine is almost gone. After adding a few drops of starch solution, continue to titrate. While

titrating, vigorously shake the conical bottle until the blue color disappears. Record the volume (V_1) of sodium thiosulfate consumed at this time and measure the volume of empty solution consumed, V_0.

$$\text{Iodine value} = \frac{(V_0 - V_1) \times C \times 12.69}{m}$$

V_0-volume of titrant, mL of blank
V_1-volume of titrant, mL of sample
C- normality of Na2 S2O3 solution
m- mass, grams of sample

Saponification value

Accurately weigh 2 g of samples in a conical bottle, add 25 mL of sodium hydroxide-ethanol solution, add boiling aid, connect the reflux condensing tube to the conical bottle, and put the conical bottle on the heating device to slowly boil and shake, and maintain the boiling state of the oil for 60 minutes. Then, phenolphthalein indicator (0.5~1mL) was added and titrated with standard solution of hydrochloric acid until the pink color disappeared. Record the volume (V_1) of sodium thiosulfate consumed and no sample was added as a control.

$$\text{Saponification value} = \frac{(V_0 - V_1) \times C \times 56.1}{m}$$

V_0- volume, mL of standard solution of hydrochloric acid consumed by the blank
V_1- volume, mL of standard solution of hydrochloric acid consumed by the sample
C- normality of standard solution of hydrochloric acid, mol/L
m- mass, grams of sample
